# Supplementary material for: A transient enhancement of Mercury’s exosphere at extremely high altitudes inferred from pickup ions
Source: Nat Commun. 2020 Sep 29;11:4350. doi: 10.1038/s41467-020-18220-2 (PMC7525509; doi:10.1038/s41467-020-18220-2)
Supplement: Supplementary file 2 — Reporting Summary [file 41467_2020_18220_MOESM2_ESM.pdf]

nature research

Corresponding author(s): Jamie M. Jasinski

Last updated by author(s): May 25, 2020

Reporting Summary

Nature Research wishes to improve the reproducibility of the work that we publish. This form provides structure for consistency and transparency in reporting. For further information on Nature Research policies, seeAuthors & Referees and theEditorial Policy Checklist .

Statistics

For all statistical analyses, confirm that the following items are present in the figure legend, table legend, main text, or Methods section.

n/a	Confirmed	
x	☐	The exact sample size (*n*) for each experimental group/condition, given as a discrete number and unit of measurement
x	☐	A statement on whether measurements were taken from distinct samples or whether the same sample was measured repeatedly
x	☐	The statistical test(s) used AND whether they are one- or two-sided<br>*Only common tests should be described solely by name; describe more complex techniques in the Methods section.*
x	☐	A description of all covariates tested
x	☐	A description of any assumptions or corrections, such as tests of normality and adjustment for multiple comparisons
x	☐	A full description of the statistical parameters including central tendency (e.g. means) or other basic estimates (e.g. regression coefficient) AND variation (e.g. standard deviation) or associated estimates of uncertainty (e.g. confidence intervals)
x	☐	For null hypothesis testing, the test statistic (e.g. *F*, *t*, *r*) with confidence intervals, effect sizes, degrees of freedom and *P* value noted<br>*Give P values as exact values whenever suitable.*
x	☐	For Bayesian analysis, information on the choice of priors and Markov chain Monte Carlo settings
x	☐	For hierarchical and complex designs, identification of the appropriate level for tests and full reporting of outcomes
x	☐	Estimates of effect sizes (e.g. Cohen's *d*, Pearson's *r*), indicating how they were calculated

Our web collection on statistics for biologists contains articles on many of the points above.

Software and code

Policy information about availability of computer code

Data collection	Data was collected by a NASA spacecraft, and made available online by the MESSENGER mission Science Team on the Planetary Data System (PDS).
Data analysis	Data was plotted using IDL, powerpoint (photoshop was used for an illustration). Data and code availability is described in the Data and Code availability section. All the data, and all the results are provided in a supplementary table (an excel spreadsheet).

For manuscripts utilizing custom algorithms or software that are central to the research but not yet described in published literature, software must be made available to editors/reviewers. We strongly encourage code deposition in a community repository (e.g. GitHub). See the Nature Research guidelines for submitting code & software for further information.

Data

Policy information about availability of data

All manuscripts must include a data availability statement. This statement should provide the following information, where applicable:

- Accession codes, unique identifiers, or web links for publicly available datasets
- A list of figures that have associated raw data
- A description of any restrictions on data availability

All the data used in this study can be found at NASA's Planetary Data System (https://pds.jpl.nasa.gov). Data and results presented in the Figures can be found in the Source Data File, in the form of data tables. No code is provided. Figures were created using IDL and Powerpoint.

Field-specific reporting

Please select the one below that is the best fit for your research. If you are not sure, read the appropriate sections before making your selection.

Ecological, evolutionary & environmental sciences study design

All studies must disclose on these points even when the disclosure is negative.

Study description	MESSENGER was a spacecraft with a variety of instrumentation that orbited Mercury in 2011-2015.
Research sample	We use one event in the MESSENGER plasma observations - where we use the magnetometer (MAG) and the Fast-Imaging Plasma Spectrometer (FIPS) to analyse a ~9minute long event.
Sampling strategy	n/a
Data collection	Data was collected by a NASA spacecraft - the MESSENGER spacecraft.
Timing and spatial scale	MAG was a fluxgate magnetometer. It was mounted on a 3.6m long boom. It had a resolution of 0.047 nT and a maximum time resolution of 20 vectors per second.<br><br>FIPS was a time-of-flight (TOF) mass spectrometer. The instrument measured ions with a range in energy-per-charge of 46 eV – 13 keV/e, a range in mass-per-charge of 1 – 60 amu/e, and a time resolution of ~8s. The angular resolution was ~15°. The effective field of view of FIPS was ~1.15 π steradian as 0.25 π steradian was blocked by the spacecraft sunshade. Because FIPS does not have a 4π steradian field of view, unless the boresight of the instrument is pointing close to the solar wind ram direction, solar wind plasma will not be observed (as was occurring during this event). The signal-to-noise of heavy ions is improved by grouping heavy planetary ions into two groups: the Na+ group (m/e = 21–30amu/e, including Na+, Mg+, and Si+), and the O+ group (m/e = 16–20amu/e, including O+ and water-group ions).
Data exclusions	n/a
Reproducibility	All the data is available publicly.
Randomization	n/a
Blinding	n/a

Did the study involve field work? ☐ Yes ☒ No

Reporting for specific materials, systems and methods

We require information from authors about some types of materials, experimental systems and methods used in many studies. Here, indicate whether each material, system or method listed is relevant to your study. If you are not sure if a list item applies to your research, read the appropriate section before selecting a response.

Materials & experimental systems

n/a	Involved in the study
☒	☐ Antibodies
☒	☐ Eukaryotic cell lines
☒	☐ Palaeontology
☒	☐ Animals and other organisms
☒	☐ Human research participants
☒	☐ Clinical data

Methods

n/a	Involved in the study
☒	☐ ChIP-seq
☒	☐ Flow cytometry
☒	☐ MRI-based neuroimaging
